# Cortical Power-Density Changes of Different Frequency Bands in Visually Guided Associative Learning: A Human EEG-Study

**DOI:** 10.3389/fnhum.2018.00188

**Published:** 2018-05-08

**Authors:** András Puszta, Xénia Katona, Balázs Bodosi, Ákos Pertich, Diána Nyujtó, Gábor Braunitzer, Attila Nagy

**Affiliations:** ^1^Sensorimotor Lab, Department of Physiology, Faculty of Medicine, University of Szeged, Szeged, Hungary; ^2^Laboratory for Perception & Cognition and Clinical Neuroscience (LPCCN), National Institute of Psychiatry and Addictions at Nyírő Gyula Hospital, Budapest, Hungary

**Keywords:** EEG, acquired equivalence, associative learning, FFT, time-frequency analysis

## Abstract

The computer-based Rutgers Acquired Equivalence test (RAET) is a widely used paradigm to test the function of subcortical structures in visual associative learning. The test consists of an acquisition (pair learning) and a test (rule transfer) phase, associated with the function of the basal ganglia and the hippocampi, respectively. Obviously, such a complex task also requires cortical involvement. To investigate the activity of different cortical areas during this test, 64-channel EEG recordings were recorded in 24 healthy volunteers. Fast-Fourier and Morlet wavelet convolution analyses were performed on the recordings. The most robust power changes were observed in the theta (4–7 Hz) and gamma (>30 Hz) frequency bands, in which significant power elevation was observed in the vast majority of the subjects, over the parieto-occipital and temporo-parietal areas during the acquisition phase. The involvement of the frontal areas in the acquisition phase was remarkably weaker. No remarkable cortical power elevations were found in the test phase. In fact, the power of the alpha and beta bands was significantly decreased over the parietooccipital areas. We conclude that the initial acquisition of the image pairs requires strong cortical involvement, but once the pairs have been learned, neither retrieval nor generalization requires strong cortical contribution.

## Introduction

**Figure 1:**
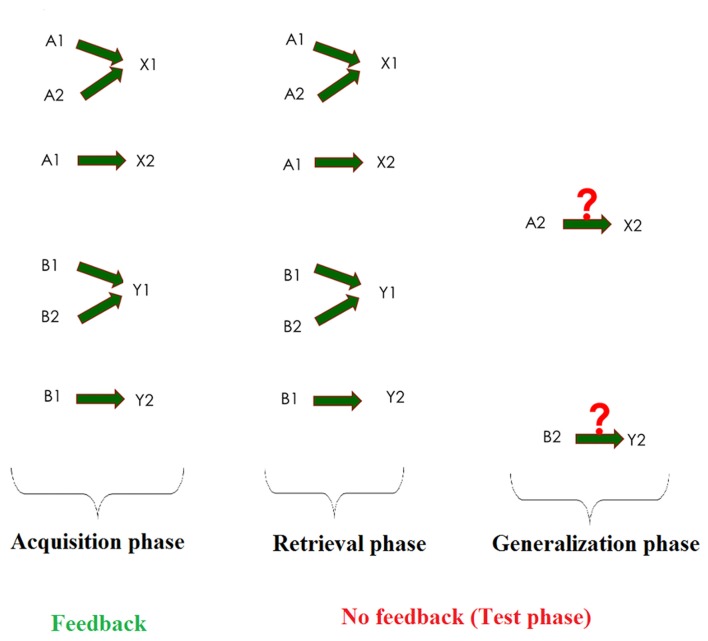
Summary of the acquired equivalence task. The task consists of three phases: acquisition, retrieval and generalization. In the acquisition phase, the subject has to learn three image pairs (one fish and one face make a pair) out of four pairs, one by one, through trial-and-error learning with feedback. Then, in the test phases, the subject will get no feedback, and, beyond the already learned three pairs (retrieval), a new, previously not taught but predictable pair is also presented (generalization).

Associative learning is a basic cognitive function, through which discrete and often strongly different ideas and percepts are linked together. This type of learning is responsible for classical conditioning (Ito et al., 2008), as well as weather-prediction (Gluck et al., 2002), latent inhibition (Weiss and Brown, 1974) and sensory preconditioning (Rescorla, 1980). Visual equivalence learning is a special kind of associative learning, which can be tested with the Rutgers Acquired Equivalence Test (RAET, Myers et al., 2003). The RAET can be divided into two main phases. The first one of these is the acquisition phase where the subjects learn to associate two different visual stimuli. The participants’ task throughout the whole test is to indicate their choice by pressing one of two marked keyboard buttons. During the acquisition phase, the computer provides feedback about the correctness of the responses. When this phase is over, the test phase follows. In this phase, both the previously learned stimulus pairs (retrieval) and hitherto not seen but predictable associations (generalization or transfer) are presented. The subjects get no feedback about the correctness of their responses in the test phase (Figure 1).

Optimal performance in the acquisition phase appears to depend mainly on the integrity of the basal ganglia, whereas the test phase performance (both retrieval and generalization) has been linked to the integrity of the hippocampal region (Myers et al., 2003; Moustafa et al., 2009). Clinical studies corroborate this. Patients with Alzheimer’s and schizophrenia, characterized by hippocampal functional deficit (Seab et al., 1988; Altshuler et al., 1998) showed intact acquisition but poor retrieval and generalization as compared to healthy controls (Bódi et al., 2009; Weiler et al., 2009). On the other hand, patients with Parkinson’s, affecting primarily the basal ganglia (Montgomery, 2009), performed poorly in the acquisition phase. However, if they managed to pass it, their retrieval and transfer performance was comparable to controls (Myers et al., 2003; Ventre-Dominey et al., 2016). In a recent study of ours, we demonstrated altered performance in migraine in both the acquisition and test phases of RAET, pointing to suboptimal functioning of the basal ganglia in migraine (Öze et al., 2017).

As previous investigations emphasized the role of the hippocampus and the basal ganglia during the test, (Moustafa et al., 2010) information is lacking about the cortical areas involved in RAET in healthy humans. Earlier studies indicated that increased gamma band phase coherence over parieto-occipital areas is important during associative learning (Miltner et al., 1999; Gruber et al., 2001), and that frontal midline theta power elevation is related to working memory maintenance and retrieval (Hsieh and Ranganath, 2014; Kardos et al., 2014). In a test where the subjects had to learn categories, the contribution of the prefrontal associative cortex was demonstrated (Helie et al., 2015), but hitherto the cortical contribution to acquired equivalence learning has not been studied.

In this study, we sought to investigate what cortical areas are activated during the individual phases of RAET by means of multichannel EEG recordings in healthy human subjects. We hypothesized that activation would be seen in the associative cortices (i.e., the prefrontal and parieto-temporo-occipital regions) that serve as the cortical input to the cognitive loops of the basal ganglia (Shepherd, 2003). Specifically, we hypothesized that different activation patterns would be seen in the different phases of the paradigm, as what we know so far is that the different phases are related to different subcortical structures, which suggests different cortical input sources.

## Materials and Methods

### Participants

EEG data of 30 healthy young adults were recorded. The data of six participants were excluded because of bad signal quality. Thus, we present the results of 24 participants (14 females, 10 males, mean age: 26 ± 5.28 years). The participants were free of any ophthalmological or neurological conditions. The participants were recruited on a voluntary basis from our university. The potential subjects were informed about the background and goals of the study, as well as about the procedures involved. It was also emphasized that given the lack of compensation or any direct benefit, the participants were free to quit at any time and without any consequence (no one did so). This study was carried out in accordance with the recommendations of the Guideline for non-invasive investigations involving healthy human volunteers, of the Medical Ethics Committee of the University of Szeged, with written informed consent from all subjects and also in accordance with the Declaration of Helsinki. The protocol was approved by the Medical Ethics Committee of the University of Szeged Hungary. The datasets generated and analyzed during the current study are available from the corresponding author on request.

### The Instrument

The testing software (described in Myers et al., 2003 and originally written for iOS) was adapted to Windows and translated into Hungarian in Assembly for Windows, with the written permission of the copyright holder. The paradigm was also slightly modified to make getting through its acquisition phase by mere guessing less probable (see below). The tests were run on a PC. The stimuli were displayed on a standard 17″ CRT monitor (refresh rate 60 Hz) in a quiet room separated from the recording room by a semi-transparent mirror. Participants sat at a 114-cm distance from the monitor. One participant was tested at a time and no time limit was set. The test was structured as follows: On each trial of the task, participants saw a face and a pair of fish (where each member of the pair had different color), and had to learn through trial and error which fish was associated with which face (Figure 1).

There were four faces (A1, A2, B1, B2) and four possible fish (X1, X2, Y1, Y2), referred to as antecedents and consequents, respectively. In the initial (acquisition) stages, the participants were expected to learn that when A1 or A2 appeared, the correct answer was to choose fish X1 over fish Y1; given face B1 or B2, the correct answer was to choose fish Y1 over fish X1. In that context, if the associations are successfully learned, participants also learn that face A1 and A2 are equivalent with respect to the associated fish (faces B1 and B2 likewise). Next, participants learned a new set of pairs: given face A1, they had to choose fish X2 over Y2, and given face B1, fish Y2 over X2. This was the end of the acquisition phase. Until this point, the computer had provided feedback about the correctness of the choices, and six of the possible eight fish-face combinations had been taught to the participants. In the following phase (the test phase), no feedback was provided anymore, but beside the already acquired six pairs (retrieval testing) the hitherto not shown last two pairs were also shown (generalization testing). Having learned that faces A1 and A2 are equivalent, participants may generalize from learning that if A1 goes with X2, A2 also goes with X2; the same holds true for B2 (equivalent to B1) and Y2 (associated with B1). While the formal description may make the impression that the task is a difficult one, in fact, healthy children (Goyos, 2000) and also mentally retarded individuals (de Rose et al., 1988; Dube et al., 1989) reliably make this kind of generalization. During the acquisition stages, new associations were introduced one by one, mixed with trials of previously learned associations. To be allowed to proceed, the subjects had to reach a predefined number of consecutive correct responses after the presentation of each new association (four after the presentation of the first association, and 4, 6, 8, 10, 12 with the introduction of each new association, respectively). This meant an elevated number of the required consecutive correct responses compared to the original paradigm, which made getting through the acquisition phase by mere guessing less probable. Similarly, in the test phase there were 48 trials (12 trials of new and 36 trials of previously learned associations), as opposed to the 16 trials of the original paradigm.

### Data Acquisition

Sixty-four channel EEG recordings were made. Data were acquired in Actiview, via the ActiveTwo AD-box with 64 active electrodes (Biosemi B.V., Netherlands). The signal of each electrode was referenced to the algebraic sum of the electric signals recorded by five scalp electrodes given by the manufacturer (FPz, T7, Cz, T8, Oz). The sampling rate was 2048 Hz. The impedance of the electrodes was consistently below 5 kΩ. Raw signals were recorded on the stimulating computer. The stimulating software generated trigger signals (TTL pulses) to indicate the beginning of each trial. These trigger signals were recorded on an additional (65th) channel. To obtain baseline activity, 1-min-long resting state activities were recorded before and after stimulus presentation.

### Data Analysis

**Figure 2:**
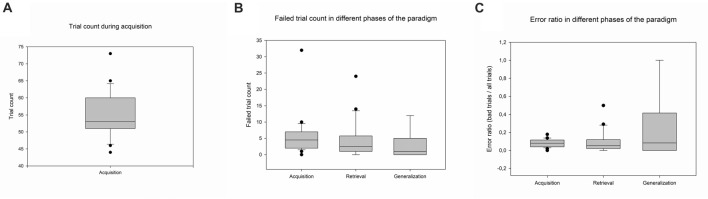
Psychophysical results. Panel **(A)** denotes the number of trials in the acquisition phase. Panel **(B)** shows the number of failed trials in different phases of the paradigm, and Panel **(C)** shows the error ratio during all three phases of the paradigm. The lower margin of the boxes indicates the 25th percentile, the line within the boxes marks the median, and the upper margin of the box indicates the 75th percentile. Whiskers (error bars) above and below the box indicate the 90th and 10th percentiles.

The psychophysical data were analyzed in three groups: data from the acquisition phase, data from the retrieval part of the test phase (i.e., when the participant was presented with already learned associations) and data from the generalization part of the test phase (i.e., when the participant was presented with previously not learned associations). The number of correct and wrong responses were calculated in all phases, as well as the ratio of these to the total number of trials during the respective phase (Figure 2). The number of trials necessary for the completion of the acquisition phase was also recorded.

### Preprocessing

After visual inspection to confirm that the signal-to-noise ratio was acceptable, the raw EEG data were first exported to a .mat file using Spike2 (CED). This was followed by high-pass filtering (>2 Hz, FIR filter). The signal of each channel was referenced to the average signal of all channels. All trials were visually inspected and those containing EMG or other artifacts not related to blinks were manually removed. The removal of blink/oculomotor artifacts was based on independent components analysis performed in the Eeglab toolbox for Matlab (Delorme and Makeig, 2004). The Eeglab toolbox was also used to interpolate noisy channels. Then we used Laplacian to improve the spatial resolution of the recording (Perrin et al., 1989). Finally, the trials were sorted by the phases of the psychophysical paradigm (acquisition, retrieval and generalization), based on the trigger signals and on the event file generated by the stimulating software.

### Fast Fourier Transformation

The majority of the trials were somewhat longer than 1 s. To avoid mismatch on summation, the first second of each trial (2048 data points) was analyzed. If the trial was shorter than 1 s (data points <2048), the trial was not analyzed to avoid zero-padding artifacts during the Fast Fourier Transform (FFT). The baseline periods (1 min) were divided into 1-s-long (2048 data points) epochs. After these pre-processing steps, FFT was performed on all trials in each phase, and for every channel. To give an example, in the acquisition phase the power spectra were whitened and normalized to baseline as follows:

$$N_{fr}\;=\;100\;+\;100*\left(\prod_{i=1}^{n}PA_{fr_{i}}-\prod_{i=1}^{n}PB_{fr_{i}}\right)/\prod_{i=1}^{n}PBfr_{i}$$

where *N* is the normalized power density of a given *fr* frequency band for a given channel, *PA* is the whitened power density in the acquisition phase’s given *i* trial within the same channel and same *fr* frequency, and *PB* is the whitened power density during baseline activity. Note that both for *PB* and *PA*, the letter *n* indicates the number of trials within the phase that was compared to the baseline (in this case the acquisition phase). As the baseline activity was longer than the compared periods (i.e., the signal belonging to a given phase), the baseline activity was cropped to match the given phase by cutting 1-s-long periods randomly.

After the FFT, the nonparametric permutation test was utilized to compare power spectra among the different phases of the behavioral task, in the following frequency bands: delta (1–4 Hz), theta (4–8 Hz), alpha (9–14 Hz), beta (15–31 Hz) and gamma (32–70 Hz). The following comparisons were made: baseline-acquisition phase, baseline-retrieval phase and baseline-generalization phase, learning phase-retrieval phase, learning phase–generalization phase, retrieval phase–generalization phase. Statistically significant differences were tested based on nonparametric permutation testing and correction for multiple comparisons at the minimum-maximum point of the null-hypothesis distribution.

The data set for the global band was generated by iteratively calculating the mean difference of randomized permutation of the power values of a particular channel in a given frequency band in two different phases of the paradigm. The *Z*-scores for each channel were then calculated between the distributions derived from the global band and the mean difference of the power values in a given frequency band between the two different analyzed phases. *Z*-scores were corrected by the minimum and maximum point of the null hypothesis distribution, also known as cluster mass statistics (Ing and Schwarzbauer, 2014). Group-level analysis of the FFT was carried out in the same way as in the individual analysis described above, with the difference that the random permutation was performed across the mean power values of the subjects and not across the power value of each individual trial.

For the FFT topographical plots, we used the “*topoplot”* function of EEGlab (Delorme and Makeig, 2004).

### Morlet Wavelet Convolution

Time-frequency analysis was performed using continuous Morlet Wavelet Convolution (CMW) via FFT algorithm (Cohen, 2014). The convolution was decomposed into different steps. First, we performed FFT on one selected channel of the raw data. Then, we created complex Morlet wavelets for each frequency (1–70 Hz) on which we executed the FFT. After that, we calculated the dot product of the given channel’s FFTs and the FFTs of the complex Morlet wavelets at each individual frequency, which yielded 70 complex numbers. An inverse FFT of the dot product results was utilized to show power alterations in the time domain as follows:

$$K_{x}\;=\;IFFT\left(fft(C)\cdot fft(W_{x})\right)$$

where the *K* is the time-series of the given channel, wavelet-filtered to *x*-frequency, *C* is the time series of all trials of different phases, and *W* is the complex Morlet wavelet at a given × frequency. To avoid the edge-artifacts of the Morlet wavelet convolution, the raw data was multiplied five times before the convolution, yielding a two-series-long buffer zone at the beginning and the end of the time-series, which were cut out after the time-frequency analysis. After that, the data were cut according to the different phases of the paradigm (baseline, acquisition, retrieval, generalization). The data set for the global band was generated by iteratively calculating the mean difference of randomized permutation of the power values of a particular channel in a given frequency band in two different phases of the paradigm. The *Z*-scores for each channel were then calculated between the distributions derived from the global band and the mean difference of the power values in a given frequency band between the two different analyzed phases. *Z-scores* were corrected by the minimum and maximum point of the null hypothesis distribution. See individual time-frequency plots in Supplementary Figure S1. Group-level analysis of the CMW was carried out in the same way as in the individual analysis described above, with the difference that the random permutation was performed across the mean power values of the subjects and not across the power value of each individual trial. These procedures are shown in detail in the Supplementary Material (Supplementary Figure S2).

### Correlation Between Performance in the Psychophysical Test and Power Density Changes

Correlation between individual performance and power density changes in a given channel and frequency band was also calculated in each phase of the paradigm. Performance was defined as the ratio of the successful trials to all trials. Individual power changes were expressed as the individual *Z*-scores between the baseline activity’s power density and the given phase’s power density in a given channel in a given frequency band. Pearson correlation coefficients were calculated using the *“corr”* function of Matlab. The *t*-score for each correlation coefficient was calculated as follows:

$$t_{ch,fr}\;=\;r_{ch,fr}*\sqrt{\frac{n\;-\;2}{1\;-\;r_{ch,fr}^{2}}}$$

where *r* is the correlation coefficient in channel *ch* and *fr* frequency band, *n* is the number of samples (in this case it was 24), and the *t* is the calculated *t*-value in given channel and frequency band. *T*-values whose absolute value were smaller than 2.819 (which is the critical *t*-value if the degree of freedom is 22 and the significance level is 0.01) were set to 0. The corrected *t*-values in different frequency bands in each phase of the paradigm were plotted to a topographical map using EEGLab “*topoplot”* function.

### Data Visualization

**Figure 3:**
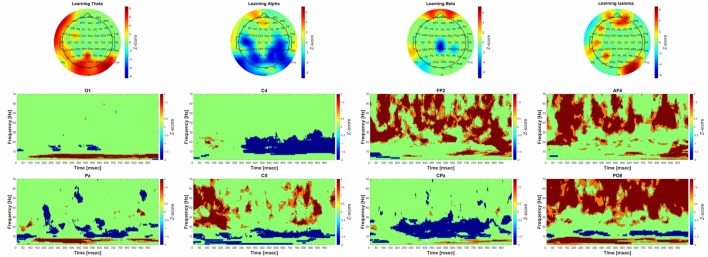
Significant group-level changes in the power of the different frequency bands during acquisition. The power of the investigated four frequency bands (theta, alpha, beta, gamma) during acquisition were compared to the baseline activity using nonparametric permutation test with correction for multiple comparisons at the minimum-maximum point of the null-hypothesis distribution. The upper part of the figure shows the Fast Fourier Transform (FFT) results of the 64 channel EEG recordings in topographic representation in different frequency band during acquisition, while the lower part of the figure corresponds to the group-level time-frequency results in different channels (O1, C4, FP2, AF4, Pz C5, CPz, PO8, respectively). The color scales beside the upper three panels indicate the *z*-score values, obtained by calculating cluster-mass statistics in individual power changes between acquisition and baseline activity. The red color indicates significant power-increase and the blue color represents significant power-decrease compared to baseline. The *z*-scores threshold was set to 1.645 (which is equal to the 0.05 *p*-value).

**Figure 4:**
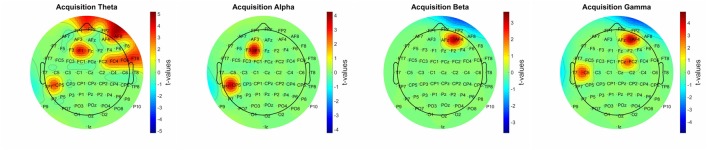
Significant power-performance correlation during acquisition in different frequency bands. The corrected *t*-values in different frequency bands were plotted to a topographical map using EEGLab “*topoplot”* function.

**Figure 5:**
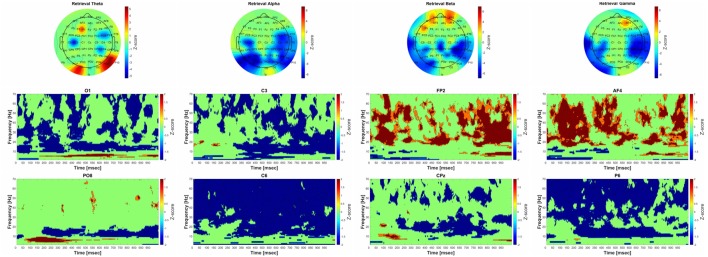
Significant group-level changes in the power of the different frequency bands during retrieval. The power of the investigated four frequency bands (theta, alpha, beta, gamma) during retrieval were compared to the baseline activity using nonparametric permutation test with correction for multiple comparisons at the minimum-maximum point of the null-hypothesis distribution. The upper part of the figure shows the FFT results of the 64 channel EEG recordings in topographic representation in different frequency band during retrieval, while the lower part of the figure corresponds to the group-level time-frequency results in different channels (O1, C3, FP2, AF4, PO8 C6, CPz, P6, respectively). The color scales beside the upper three panels indicate the *z*-score values, obtained by calculating cluster-mass statistics in individual power changes between retrieval and baseline activity. The red color indicates significant power-increase and the blue color represents significant power-decrease compared to baseline. The *z*-scores threshold was set to 1.645 (which is equal to the 0.05 *p*-value).

**Figure 6:**
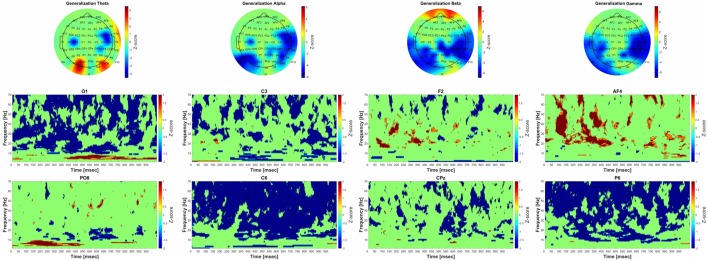
Significant group-level changes in the power of the different frequency bands during generalization. The power of the investigated four frequency bands (theta, alpha, beta, gamma) during generalization were compared to the baseline activity using nonparametric permutation test with correction for multiple comparisons at the minimum-maximum point of the null-hypothesis distribution. The upper part of the figure shows the FFT results of the 64 channel EEG recordings in topographic representation in different frequency band during generalization, while the lower part of the figure corresponds to the group-level time-frequency results in different channels (O1, C3, F2, AF4, PO8 C6, CPz, P6, respectively). The color scales beside the upper three panels indicate the *z*-score values, obtained by calculating cluster-mass statistics in individual power changes between generalization and baseline activity. The red color indicates significant power-increase and the blue color represents significant power-decrease compared to baseline. The *z*-scores threshold was set to 1.645 (which is equal to the 0.05 *p*-value).

The electrophysiological results are presented below for each phase of the paradigm (Acquisition, Retrieval and Generalization) and for the four different frequency bands (theta (4–8 Hz), alpha (9–14 Hz), beta 15–31 Hz), and gamma (32–70 Hz). The results of FFT and CMW are shown at the population level, where each phase of the paradigm (acquisition, retrieval and generalization) are compared to the baseline activity. As our research is more exploratory than hypothesis-driven we will not discuss the statistical *p*-values in detail, but we do discuss the significant *z*-score maps of the group-level statistic of the FFT and CMW. Time-frequency plots of the channels where the FFT results showed significant changes are presented for each phase of the paradigm. Examples for individual time-frequency plots can be found in the Supplementary Material (Supplementary Figure S1). The correlation between performance in the psychophysical test and cortical power changes (rho values) for each frequency band are plotted in topographical figures. See corresponding Figures 3–6 for electrophysiological results in each frequency band.

## Results

The data of 24 participants were analyzed. The participants accomplished the acquisition phase in a mean of 55 trials (SD ± 6.93, Range: 44–73). The mean number of failed trials in the acquisition phase was 4.43 (SD ± 2.67, Range: 0–32), in the retrieval phase 4.69 (SD ± 5.96, Range: 0–24), and 2.87 in the generalization phase (SD ± 4.18, Range: 0–12). The means of the error ratios in the different phases were the following: in the acquisition phase 0.08 (SD ± 0.036; Range: 0–0.14), in the retrieval phase: 0.15 (SD ± 0.17, Range: 0–0.65), and in the generalization phase 0.31 (SD ± 0.38, Range: 0–1) (Figure 2).

### Acquisition Phase

#### Power Density Changes

##### Theta Band (4–8 Hz)

The group-level analysis revealed significant power-elevation in parietooccipital-occipital areas as well as in the frontal areas. The time-frequency results indicate that the power elevation in the occipital and parietooccipital areas started earlier (around 75 ms after the beginning of the trial), than over the frontal areas (around 550 ms after the beginning of the trial).

##### Alpha Band (9–14 Hz)

A significant decrease in power could be observed during acquisition compared to baseline over the parietal, parietooccipital and temporal areas. The time-frequency results show that the power decrement was phasically present over the parietooccipital areas, and over the temporal-central areas it started around 300 ms after the beginning of the trial.

##### Beta Band (15–30 Hz)

A significant decrement of power was found during acquisition compared to baseline over the central areas. A significant increase of power was also found over frontal areas. The Morlet wavelet convolution showed that the frontal power increase occurred phasically, and the power decrease—along with the changes in the alpha frequency band—started around 300 ms after the beginning of the trial.

##### Gamma Band: (31–70 Hz)

The group-level analysis revealed significant power-elevation over the frontal, parietooccipital, and temporal areas. The results of the time-frequency analysis indicate that the power increase over the parietooccipital areas began immediately after the beginning of the trial and 50 ms later over the frontal areas.

#### Power-Performance Correlation

Significant correlation was found over the frontal and temporal areas in all frequency bands (Figure 4). The most prominent changes occurred in the theta and gamma frequency bands, while in the beta frequency band the correlation was limited to channel AF4.

### Retrieval Phase

#### Power Density Changes

##### Theta Band (4–8 Hz)

The group-level analysis revealed significant power elevation over the parietooccipital and occipital and frontal areas. The time-frequency results indicate that the power elevation over the occipital and parietooccipital areas started earlier (around 75 ms after the beginning of the trial) than over the frontal areas (around 700 ms after the beginning of the trial).

##### Alpha Band (9–14 Hz)

Significant power decrease could be observed compared to baseline over the parietal, parietooccipital and temporal areas. The results of the time-frequency analysis show that the power decrement was phasically present over the parietooccipital areas, while over the temporal-central areas it started around 300 ms after the beginning of the trial.

##### Beta Band (15–30 Hz)

There was a significant power decrease over the parietooccipital, temporal and central areas during retrieval phase compared to baseline. Significant power increase was also found over the frontal areas. CMW showed that the frontal power increase occurred phasically, and the power decrease—similarly to the changes in the alpha frequency band—started around 300 ms after the beginning of the trial.

##### Gamma Band: (31–70 Hz)

The group-level analysis revealed significant power decrease over the parietooccipital and temporal areas. Significant power increase was also found over the frontal areas. The time-frequency analysis revealed that the power changes occurred over the parietooccipital areas immediately after the stimulus onset, while over the frontal areas they began 50 ms after the beginning of the trial.

#### Power-Performance Correlation

The correlation between the performance and the power changes was not significant in either frequency band or channel.

### Generalization Phase

#### Power Density Changes

##### Theta Band (4–8 Hz)

The group-level analysis revealed significant power elevation over the parietooccipital and occipital areas, and power decrease over the temporal areas. The time-frequency analysis showed that the power elevation over the occipital and parietooccipital areas started earlier (around 75 ms after the beginning of the trial) than the central-temporal power decrease (around 300 ms after the beginning of the trial).

##### Alpha Band (9–14 Hz)

Significant power decrease could be observed compared to baseline over the parietal, parietooccipital and temporal areas. The time-frequency results show that the power decrement was phasically present over the parietooccipital areas, and over the temporal-central areas it started around 300 ms after the beginning of the trial.

##### Beta Band (15–30 Hz)

A significant power decrease compared to baseline was observed over the parietooccipital, temporal and central areas. Significant power increase was found over the frontal areas. CMW showed that the frontal power increase occurred phasically, and the power decrease—along with the changes in alpha frequency band—started around 300 ms after the beginning of the trial.

##### Gamma Band: (31–70 Hz)

The group-level analysis revealed significant power decrease over the parietooccipital and temporal areas. The results of the time-frequency analysis indicate that the power changes occurred over the parietooccipital areas immediately after stimulus onset, while over the frontal areas they started 50 ms after the beginning of the trial.

#### Power-Performance Correlation

No significant correlations were found in either frequency band or channel.

## Discussion

In the present study we analyzed the psychophysical performance and EEG data of 24 healthy young volunteers in a visual associative learning test. As for the behavioral performance in the psychophysical paradigm, the results were comparable to the findings of other studies using the same paradigm in adult healthy volunteers (Öze et al., 2017). Behavioral performance in this paradigm was widely investigated in healthy volunteers and patients with psychiatric and neurological disorders (Myers et al., 2008; Vadhan et al., 2008; Meeter et al., 2009; Simon and Gluck, 2013; Kostek et al., 2014). However, to our knowledge, no study so far has attempted to investigate cortical activity associated with behavioral performance.

Despite the considerable individual variability in the changes of the power spectra, characteristic patterns of power change could be identified over different cortical areas as related to the different phases of the paradigm at the population level. Correct and missed trials could not be compared, though, because of the low number of missed trials. Subtraction of the EEG signal recorded during the missed trials from all trials left the activation patterns virtually unchanged, so we decided to analyze and present both types of trials together.

In the acquisition phase, markedly increased population-level activity could be observed over the parieto-temporo-occipital areas, and somewhat weaker increase over the frontal associative areas in the theta (4–7 Hz) and the gamma frequency bands (over 30 Hz). Such a strong power increase was not found in the retrieval and generalization phases of the task. In those phases, power decrement was the dominant tendency over the same areas. That tendency was obvious not only in the alpha and beta frequency bands, but also in the gamma frequency band.

The detailed mathematical analysis showed the most robust power increment in the gamma frequency band (>30 Hz) in the parietal, parietooccipital and temporoparietal channels during the acquisition phase in most of the participants. These channels correspond to the associative cortical areas, which were mainly suppressed during the retrieval and generalization phases. The most noteworthy finding of this study is the strong difference in the power density changes between the acquisition and the test phases (i.e., retrieval and the generalization) in the gamma band. Furthermore, the frontal and prefrontal associative cortices showed activity increment in the gamma band in the acquisition phase too, and weak power elevation was found in the test phase. The strong increment in the power of the gamma band over the parieto-temporo occipital associative cortex suggests a critical role of this region in the studied task. These cortical structures, together with the connected basal ganglia (Postuma and Dagher, 2006) could be necessary for this kind of equivalence learning (Middleton and Strick, 2000; Yin and Knowlton, 2006). Nos such increment was detectable in the gamma band in the retrieval and the generalization phases. The explanation of this could be that the successfully learned associations had already been transmitted to the hippocampus, and the utilization of these does not require strong cortical contribution. Hamamé et al. (2014), applying a visual naming model in humans, found robust activity increment in the high gamma frequency band in the left hippocampus, 500 ms post-stimulus. Although the model tests long-term memory retrieval rather than associative retrieval, their findings correlate well with psychophysical (Gluck et al., 2003; Myers et al., 2003) and electrophysiological studies in primates regarding associative retrieval (Brincat and Miller, 2015). As high gamma activity can be regarded as an indicator of multi-unit spiking activity (Le Van Quyen et al., 2010), we assume that acquired equivalence learning requires cortical activation, whereas retrieval and generalization do not. In a similar test, where the task was category learning, marked contribution of the prefrontal associative cortex was demonstrated in the acquisition phase (Helie et al., 2015). Our results show not only increased power over the frontal areas but also a strong correlation between the performance and the gamma power changes during acquisition, which points to the frontal areas’ prominent role in memory encoding and rule based learning (Gruber et al., 2002; Hester et al., 2007). While we found similar power increment over the frontal areas during acquisition, it was less pronounced than that found in the cited studies. The reason for this difference may be the lower difficulty of the task we applied.

Power decrements were found in most of the participants in the alpha and beta frequency bands. These decrements occurred over the central and more characteristically over the parieto-temporo-occipital areas, similarly to what was found in other studies with different visual paradigms (Hanslmayr et al., 2005; Romei et al., 2008; Klimesch, 2012). Concerning the lowest frequencies (delta and theta bands), their role in cognitive tasks is still a matter of debate (Hanslmayr et al., 2016). The power spectrum analysis in our study revealed significant power elevation over the parietooccipital and occipital, as well as over the frontal areas. The enhanced frontal midline and parietooccipital power of the theta band during working memory encoding and retrieval is a well-known phenomenon in learning tasks (Klimesch et al., 1997; Weiss et al., 2000; Sauseng et al., 2010) and in attentional processes (Fellrath et al., 2016). Indeed, in our study we found that the frontal-midline theta power alterations are in strong correlation with task performance during acquisition, but not during the second part (retrieval and generalization) of the paradigm. This could indicate that the early part of the paradigm (acquisition) required a high level of attention, while the later parts (retrieval and generalization) did not.

In summary, the most robust cortical power changes were observed in the higher frequency bands (gamma, over 30 Hz) in the acquisition phase of the applied paradigm over the parieto-temporo-occipital associative cortex. The frontal associative areas were less involved. On the other hand, such power changes were not obvious in the retrieval and generalization phases. These findings indicate that the activation of the associative cortical areas is necessary for acquisition, but retrieval and generalization are relatively independent of cortical activation. In other words, basal ganglia-mediated learning in the given context depends on the cortical input, but once the equivalence has been acquired, the hippocampi can apply the learned and memorized information without significant cortical contribution.

## Author Contributions

AP, AN and GB designed the study and prepared the main manuscript text. AP, XK, ÁP and DN registered the EEG. AP analyzed the data and prepared the figures and supplementary figures. AP and BB prepared the stimuli (artwork) and programed the stimulus presentation software. All authors reviewed the manuscript.

## Conflict of Interest Statement

The authors declare that the research was conducted in the absence of any commercial or financial relationships that could be construed as a potential conflict of interest.

## Abbreviations

CMW: Complex Morlet Wavelet convolution
FFT: Fast Fourier Transform
RAET: Rutgers acquired equivalence test.
