# Supplementary material for: Cortical Power-Density Changes of Different Frequency Bands in Visually Guided Associative Learning: A Human EEG-Study
Source: Front Hum Neurosci. 2018 May 8;12:188. doi: 10.3389/fnhum.2018.00188 (PMC5951962; doi:10.3389/fnhum.2018.00188)
Supplement: Supplementary file 1 [file Image_1.pdf]

Supplementary Material

Cortical power-density changes of different frequency bands during a visually guided associative learning test: a high resolution human EEGstudy

András Puszta[1], Xénia Katona[1], Balázs Bodosi[1], Ákos Pertich[1], Diána Nyujó[1], Gábor Braunitzer[2], Attila Nagy[1*]

[1]: Department of Physiology, Faculty of Medicine, University of Szeged, Hungary

2: Laboratory for Perception & Cognition and Clinical Neuroscience (LPCCN), National Institute of Psychiatry and Addictions at Nyírő Gyula Hospital, Budapest, Hungary

*** Correspondence:** Attila Nagy
nagy.attila.1@med.u-szeged.hu

1 Supplementary Figures

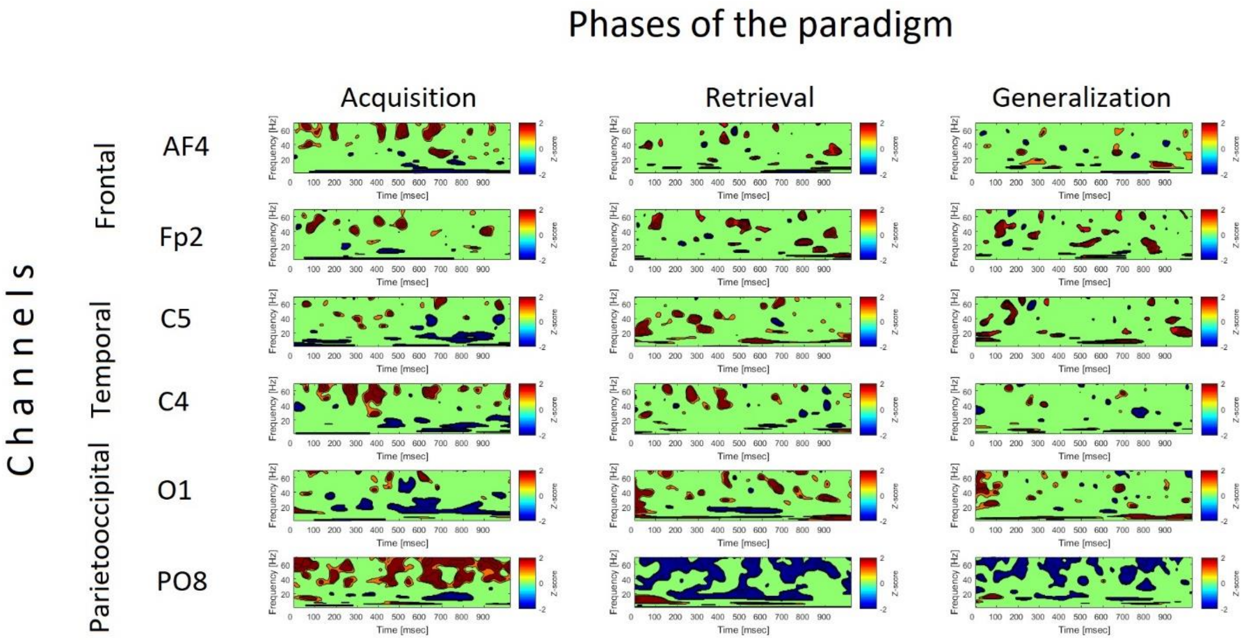

Supplementary Figure 1. Individual time-frequency results in three channels of the acquired equivalence test in different comparisons. Alterations of the frequency spectra in the time domain can be observed in different channels, between different comparisons.

Each row shows significant differences of different channels (AF4, Fp2, C5, C4, O1, PO8), while the columns represent cortical power changes during the different phases of the paradigm indicated at the upper side of the figure. The color scales beside each time-frequency plot indicate the significant power changes of the power values, which were calculated from the mean of the power changes in different

phases of the paradigm and the power of the baseline activity. The three phases (acquisition, retrieval, and generalization) were compared to the prestimulus baseline period with nonparametric permutation test with correction for multiple comparisons at the minimum-maximum point of the null-hypothesis distribution.

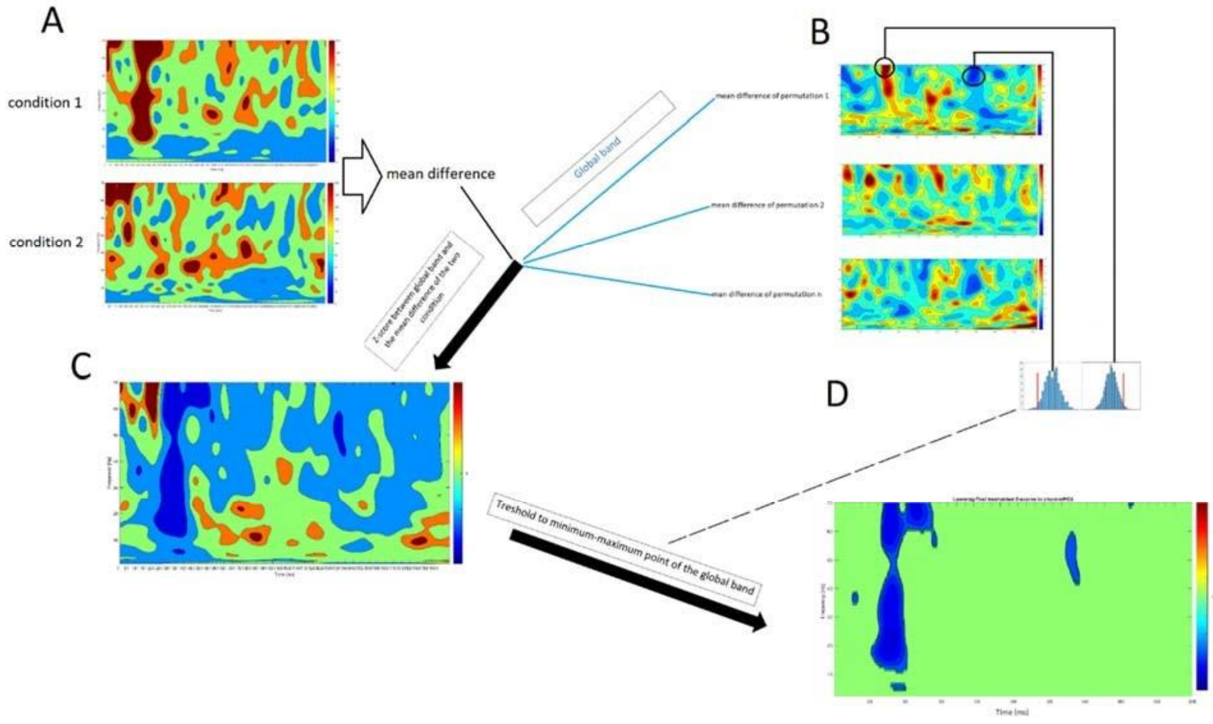

Supplementary Figure 2 Visualization of the nonparametric permutation-based test Mean difference between the two comprised phases was calculated on a given channel (A). The data set for the purposed null-hypothesis (global band) was generated by iteratively calculating the mean difference of randomized permutation of the power values of a particular channel in a given frequency band in two different phases of the paradigm (B). The Z-scores for each channel were then calculated between the distributions derived from the global band and the mean difference of the power values in a given frequency band between the two different analyzed phases(C). Z-scores were corrected by the minimum and maximum point of the null hypothesis distribution (D).
